# Phytic acid effect on periodontal ligament fibroblast: An in-vitro study

**DOI:** 10.1371/journal.pone.0295612

**Published:** 2023-12-14

**Authors:** Smriti Aryal A. C., Mohannad Nassar, Aghila Rani K. G., Ahmed M. Al-Rawi, Rania Nassar, Md. Sofiqul Islam

**Affiliations:** 1 Department of Oral and Craniofacial Health Sciences, College of Dental Medicine, University of Sharjah, Sharjah, United Arab Emirates; 2 Department of Preventive and Restorative Dentistry, College of Dental Medicine, University of Sharjah, Sharjah, United Arab Emirates; 3 Research Institute for Medical and Health Sciences, University of Sharjah, Sharjah, United Arab Emirates; 4 College of Medicine, Mohammed Bin Rashid University of Medicine and Health Sciences, Dubai Health, Dubai, United Arab Emirates; 5 Department of Operative Dentistry, RAK College of Dental Sciences, RAK Medical and Health Sciences University, Ras Al-Khaimah, United Arab Emirates; Universidade de Trás-os-Montes e Alto Douro: Universidade de Tras-os-Montes e Alto Douro, PORTUGAL

## Abstract

**Objectives:**

This study evaluated phytic acid (IP6) effect on the viability, alkaline phosphatase (ALP) activity and calcium release of human periodontal ligament (HPDL) cells in optimal (OGL) and elevated glucose level (EGL) in cell culture media.

**Materials and methods:**

Cells were seeded in OGL (1000mg/L) or EGL (4500 mg/L) media. IP6 was added at 0.005%, 0.01% or 0.02% concentrations for 24 or 48h, and XTT assay was performed. Cell differentiation and calcium release in presence of 0.02% IP6 in OGL or EGL in non-osteogenic or osteogenic media were analyzed using ALP assay and alizarin red staining, respectively.

**Results:**

In OGL, IP6 enhanced the viability of the cells at both exposure times (P<0.05). However, IP6 lowered the viability of the cells with the presence of EGL compared to the control at both exposure times, except for 0.02% IP6 which showed comparable viability to the control at 48 h. In OGL and EGL, ALP activity of the cells was not affected by the presence of IP6 in non-osteogenic media; however, in osteogenic media IP6 lowered the ALP activity. Meanwhile, calcium release was the highest with IP6 within osteogenic media of EGL.

**Conclusions:**

IP6 effects on the HPDL cells were dependent on IP6 concentration, time of exposure, glucose levels and the osteogenic condition of the media.

**Clinical relevance:**

This study gives insights on the potential therapeutic effect of IP6 as adjunctive periodontal therapy in patients with diabetes.

## Introduction

A staggering number of patients are believed to be living with diabetes around the globe, and this number is steadily increasing and expected to reach 750 million by 2045 [1]. This disease is characterized by elevated plasma glucose concentration that has a burden on most of the body’s systems especially in patients with chronically uncontrolled levels. Several types of cells are vulnerable to hyperglycemia that induces cellular injuries manifesting as long-lasting tissue damage and diabetes-related complications [2]. These complications are ascribed to inflammation, ischemia and increased reactive oxygen species, and are more pronounced at highly vascularized and innervated tissues. Thus, the oral cavity is not an exception. Most studies that evaluated the oral complications have focused on the effect on the periodontium [3]. The periodontal ligament microvasculature has been described as highly organized and dense capillary system that is required to support the functions of the periodontium to defend against microbial invasion which demands a rapid cell turnover [4].

The faster progression of periodontal disease in diabetic patients is attributed to hyperglycemia which has a direct negative impact on oral microbial composition, collagen metabolism and cellular functions [5]. The human periodontal ligament (HPDL) is immensely involved and probably the most important in the regeneration and wound healing of the periodontium due to the distinctive characteristics of the highly functionalized HPDL cells. The behavior of these cells is influenced by several factors, and culturing these cells under different conditions is crucial in order to gain insights into designing therapeutic approaches [6, 7]. The effect of high glucose levels on the cellular activity of HPDL cells is not well-established, thus it is important to further investigate the consequences and explore potential mitigative measures. High glucose level was found to disturb the differentiation and calcification of HPDL cells thus contributing, at least in part, to impaired periodontal healing and regeneration in diabetic patients [8]. Moreover, inhibited chemotactic response of HPDL cells to growth factors has been observed under elevated glucose levels due to overexpression of fibronectin receptors on cell surfaces [9]. Amplified adhesiveness of HPDL cells to the matrix proteins in the periodontium is an expected result for the overexpression of fibronectin receptor that leads to reduced ability of the cells to migrate to contribute to healing. Furthermore, the activity of cathepsins, major intracellular proteinases responsible for collagen metabolism, is suppressed by high glucose resulting in compromised protein degradation and the accumulation of extracellular matrices which is viewed as one of the complications of diabetes [10]. HPDL cells apoptosis is also induced by high glucose through a process mediated by caspase-3 [11]. Research efforts to ameliorate glucose toxicity on HPDL cells have been previously attempted with successful findings through the use of DNA methyltransferase inhibitor to suppress of methylation or by medications such as metformin which is natriuretic peptide receptor-3 antagonist [12]. However, these days more attention is given to natural bioactive materials due to various offered attractive features and the relatively high acceptance of the public to the use of these agents. Bhattarai et al. investigated the role of p‐coumaric acid in inhibiting diabetes‐associated periodontal destruction and found that supplementation with this naturally occurring acid reversed the impairment induced by high glucose levels [13].

Phytic acid or inositol hexakisphosphate (IP6), is the major storage form of phosphorus in plants seeds and bran, and omnipresent in mammalian cells to serve various biological functions. Historically, IP6 was viewed as antinutrient agent due to its immense ability to chelate with cations thus preventing proper absorption of essential minerals from food. However, this has changed over the years with the availability of compelling evidence that supports the beneficial effects that this agent has to offer due to its unique properties [14]. The antihyperglycemic activity of IP6 was studied on a rat model by Lee et al. Fasting and random glucose and hemoglobin A1c levels were lower in rats fed with IP6-rich diet compared to control. According to the authors the notable antihyperglycemic activities of IP6 create an opportunity to develop a novel class of antidiabetic agents. However, the exact mechanisms for these effects are not fully understood, as yet [15]. IP6 effects on starch digestibility, hormones, insulin signaling, and glucose metabolism have been proposed as possible pathways [16, 17]. The attentiveness to IP6 and some of its applications in dentistry have been described several decades ago, however, the interest declined until recently revived by exploring further properties that are applicable to the dental field [18, 19]. The anti-plaque and anti-calculus functions of IP6 prompted its incorporation within oral care products to act locally in the oral cavity to prevent the progression of periodontal disease [20].

To the best of our knowledge, there seems to be a lack of understanding on the systemic effect of IP6 on HPDL cells and its role in protecting the cells from glucose-induced damage. Thus, the objectives of this study were to evaluate the effect of IP6 on the viability, alkaline phosphatase (ALP) activity, and calcium release in HPDL cells in optimal (OGL) and elevated glucose levels (EGL) within cell culture media. The null hypothesis tested was that there was no effect of IP6 on the above-mentioned cellular activities of HPDL cells.

## Materials and methods

### HPDL fibroblast cell culture

Human periodontal ligament fibroblast (HPDL) cells (HUM-iCELL-m001, Shanghai/China) were cultured in Dulbecco’s Modified Eagle Media (DMEM) (Sigma-Aldrich, Burlington/USA) supplemented with 10% fetal bovine serum (FBS) (Sigma-Aldrich, Burlington/USA) and 1% of antibiotics (Sigma-Aldrich, Burlington/USA). The cells were maintained at 37 °C, in a humid atmosphere of 95% O_2_ and 5% CO_2_ during the entire experimental period. Cells on reaching confluence, were sub-cultured using 1x trypsin-ethylenediaminetetraacetic acid (trypsin–EDTA) solution (Sigma-Aldrich, Burlington/USA) and re-suspended in fresh culture media. The cell suspension was then distributed into new culture plates and further incubated. The media was replenished every two days after seeding. Cells on reaching confluence, were sub-cultured using trypsin-EDTA solution and re-suspended in fresh culture media.

### Cell viability assay

HPDL cells were cultured (5_×_10^3^ cells/well), in triplicate, in 96-well plates containing 100 μL of DMEM-elevated glucose level (EGL) (4500 mg/L) (Sigma-Aldrich, Burlington/USA) supplemented with 10% fetal bovine serum (FBS) (Sigma-Aldrich, Burlington/USA) and 1% of antibiotics (Sigma-Aldrich, Burlington/USA) or DMEM-optimal glucose level (OGL) (1000 mg/L) (Sigma-Aldrich, Burlington/USA) supplemented with 10% FBS (Sigma-Aldrich, Burlington, USA) and 1% of antibiotics (Sigma-Aldrich, Burlington/USA) and then the cells were treated with 0.005%, 0.01% or 0.02% concentrations of IP6. Cell culture in fresh media without IP6 served as the control. After 24 and 48 h, plates were analyzed by cell proliferation kit II (XTT) (Roche, Penzberg/Germany). As per the manufacturer instruction, the cells were treated with the XTT reagent/activator mix then were incubated at 37 °C for 4 h followed by measurement of absorbance at 450 nm (Biotek 800/TS, Agilent, Santa Clara/USA). The percentage cell viability was calculated based on the mean absorbance values.

### Alkaline phosphatase assay (ALP)

HPDL cells were cultured (5_×_10^4^ cells/well), in triplicate, in 12-well plates containing 1 ml of DMEM-EGL (Sigma-Aldrich, Burlington/USA) supplemented with 10% fetal bovine serum (FBS) (Sigma-Aldrich, Burlington/USA) and 1% of antibiotics (Sigma-Aldrich, Burlington/USA) or DMEM-OGL (Sigma-Aldrich, Burlington/USA) supplemented with 10% fetal bovine serum (FBS) (Sigma-Aldrich, Burlington/USA) and 1% of antibiotics (Sigma-Aldrich, Burlington/USA). When cultured cells reached its confluency the media was supplemented with or without osteogenic media DMEM-EGL(Sigma-Aldrich, Burlington/USA) supplemented with 10% fetal bovine serum (FBS) (Sigma-Aldrich, Burlington/USA) or DMEM-OGL (Sigma-Aldrich, Burlington/USA) supplemented with 10% FBS, 50 μg/mL L-ascorbic acid 2-phosphate (Sigma-Aldrich, Burlington/USA), 10 mM sodium -glycerophosphate, 1% antibiotics (Sigma-Aldrich, Burlington/USA). The cells with and without osteogenic media were treated with 0.02% IP6 and were incubated in the incubator at 37 °C in 5% CO_2_ for 14 days. Cell culture in fresh media without IP6 served as the control. The media of all groups was changed every 3 days during the culture period. ALP activity was measured using an ALP Colorimetric Assay Kit (Abcam, Cambridge/UK). Briefly, control and IP6-treated groups in the presence and absence of osteogenic media were washed with phosphate buffered saline (PBS) and lysed using ALP assay buffer. Thereafter, a total of 80 μL of the cell lysate was added to 50 μL pNPP, in a 96-well plate, and the samples were shielded from direct light for 1 h at room temperature. Following that, a 20 μL stop solution (3 N NaOH) was added to the wells and the reading was performed at 405 nm using an enzyme-linked immunosorbent acid (ELISA) plate reader (Biotek 800/TS, Agilent, Santa Clara/USA).

### Calcium release and alizarin red staining

HPDL cells were cultured at a density of (5_×_10^4^ cells/ well), in triplicate, in 12-well plates containing 1ml of DMEM-EGL (Sigma-Aldrich, Burlington/USA) supplemented with 10% fetal bovine serum (FBS) (Sigma-Aldrich, Burlington/USA) and 1% of antibiotics (Sigma-Aldrich, Burlington/USA) or DMEM-OGL (Sigma-Aldrich, Burlington/USA) supplemented with 10% fetal bovine serum (FBS) (Sigma-Aldrich, Burlington/USA) and 1% of antibiotics (Sigma-Aldrich, Burlington/USA). When cultured cells reached its confluency the media was supplemented with or without osteogenic media DMEM-EGL (Sigma-Aldrich, Burlington/USA) supplemented with 10% fetal bovine serum (FBS) (Sigma-Aldrich, Burlington/USA) or DMEM-OGL (Sigma-Aldrich, Burlington/USA) supplemented with 10% FBS, 50 μg/mL L-ascorbic acid 2-phosphate (Sigma-Aldrich, Burlington/USA), 10 mM sodium -glycerophosphate, 1% antibiotics (Sigma-Aldrich, Burlington/USA). The cells with and without osteogenic media were treated with 0.02% IP6 and were incubated at 37 °C in 5% CO_2_ for 21 days. Cell culture in fresh media without IP6 served as the control. The media of all groups was changed every 3 days during the culture period. Alizarin red staining for the control and experimental groups was evaluated for calcium release at day 21 of treatment by staining with alizarin red solution (a dye that binds to calcium salts to visualize the mineralization potential of each sample). Briefly, cells were fixed in 4% paraformaldehyde for 15 min at room temperature followed by PBS wash and stained for 30 min with 2% alizarin red solution (Sigma-Aldrich, Burlington/USA). Cells were then washed 3 times with ultrapure water and prepared for imaging using phase contrast microscopy (Olympus I×73, Tokyo/Japan) at 10x magnification. To quantify the staining, alizarin stain was extracted from the cells using 10% acetic acid solution and absorbance was measured at 405 nm using a spectrophotometer (Biotek 800/TS, Agilent, Santa Clara/USA). A standard curve was also prepared using different concentrations of the alizarin red stain [21].

### Statistical analysis

Cell viability, ALP activity and calcium release data were estimated using mean values and standard deviation and were checked for significant deviation from normality (Shapiro-Wilk test) and homoscedasticity (Levene’s test). When the normality and equality variance assumptions of the data were valid, two-way ANOVA and Tukey HSD post-hoc tests were performed and then further analyzed by one-way ANOVA and Tukey’s HSD (α = 0.05).

## Results

### Cell viability

HPDL cells were cultured in media containing either OGL (1000mg/L) or EGL (4500 mg/L) to represent physiologic and diabetic blood glucose levels, respectively. The XTT assay was employed to determine cell viability in both conditions. At OGL, two-way ANOVA showed that the factor “concentration” was significant (P < 0.001) while the factor “time” was not significant (P = 0.13). However, the interaction between the two factors was significant (P < 0.001). While at EGL, the two factors and their interaction were significant (P < 0.05).

**Fig 1:**
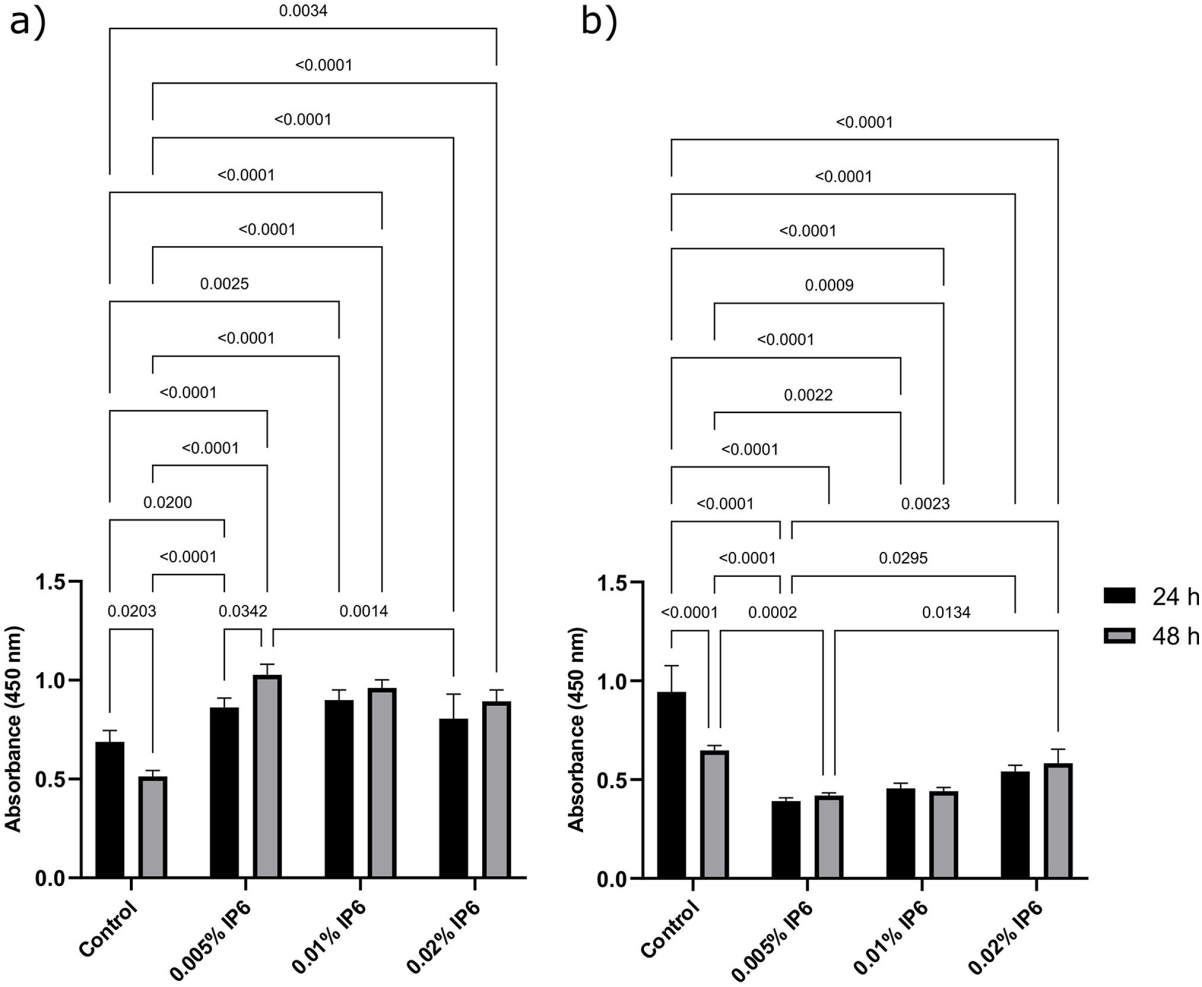
Viability of human periodontal ligament (HPDL) cells in response to phytic acid (IP6) treatment in optimal glucose level (OGL) and elevated glucose level (EGL) media. (a) XTT cell viability assay of HPDL in control and after treatment with 0.005%, 0.01% and 0.02% IP6 for 24 and 48 h in OGL media. (b) XTT cell viability assay of HPDL in control and after treatment with 0.005%, 0.01% and 0.02% IP6 for 24 and 48 h in EGL media. The results are presented as the mean ± standard deviation. Statistical analysis was performed using ANOVA.

**Fig 2:**
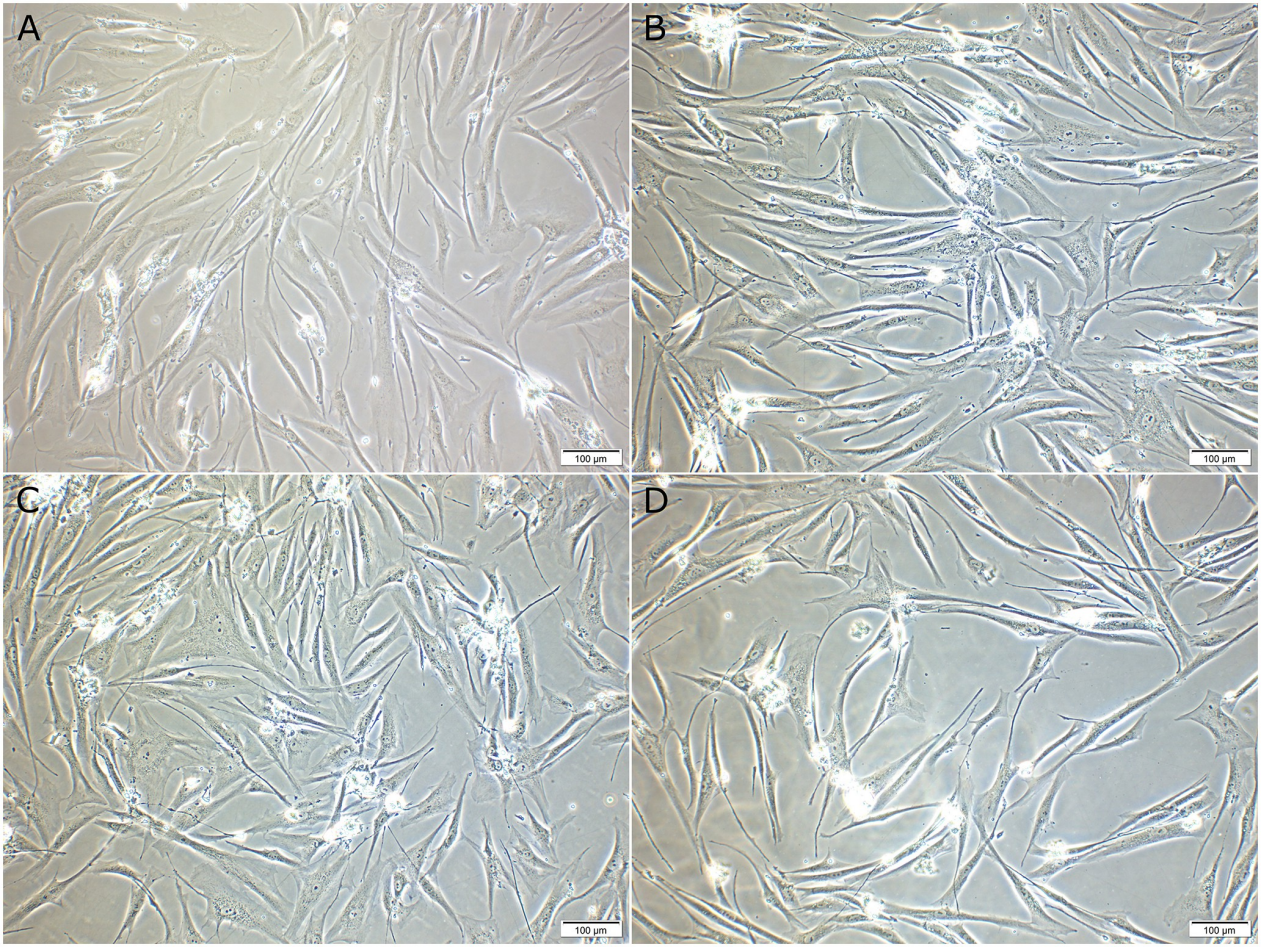
Representative light microscopy images showing human periodontal ligament (HPDL) cells at 48 h of phytic acid (IP6) treatment in optimal glucose level (OGL) media. (A) Control group (B) 0.005% IP6 (C) 0.01% IP6 (D) 0.02%. Scale bar 100μm.

**Fig 3:**
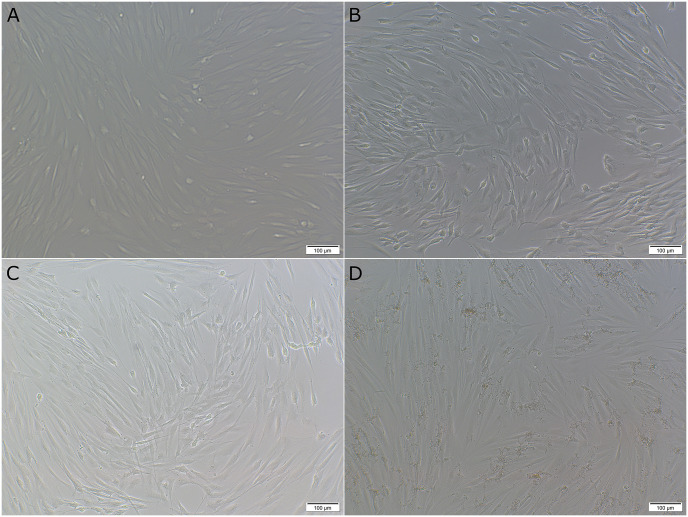
Representative light microscopy images showing human periodontal ligament (HPDL) cells at 48 h of phytic acid (IP6) treatment in elevated glucose level (EGL) media. **(A)** Control group **(B)** 0.005% IP6 **(C)** 0.01% IP6 **(D)** 0.02% IP6. Scale bar 100μm.

The effects of various concentrations of IP6 on HPDL cells’ viability in OGL and EGL are shown in Figs 1a and 2b; respectively. In cell culture media with OGL, IP6 enhanced the viability of HPDL cells at both exposure times compared to the control (P < 0.05). However, IP6 lowered the viability of the cells with presence of EGL in the media compared to the control at both exposure times (P < 0.05), except for 0.02% IP6 which showed comparable value to the control at 48 h (P > 0.05). Interestingly, intergroup comparison showed that the viability of the control group significantly dropped at 48 h compared to its counterpart at 24 h within OGL and EGL media. Meanwhile, IP6 groups showed a tendency for higher viability at 48 h in comparison to that obtained at 24 h of each counterpart group; however, this was statistically significant only for 0.005% IP6 in a media containing OGL. At each exposure time in a media with OGL, the different concentrations of IP6 resulted in comparable cell viability (P > 0.05). However, in a media with EGL, at 24h-exposure time, 0.02% IP6 resulted in higher viability compared to 0.005% IP6 (P < 0.05), while at 48 h 0.02% IP6 resulted in a viability that was significantly higher than those obtained with 0.01% and 0.005% IP6 (P > 0.05). Representative images of HPDL cells in OGL and EGL media for the control, 0.005%, 0.01% and 0.02% IP6 groups at 48 h are shown in Figs 2A–2D and 3A–3D, respectively.

### ALP activity

HPDL cells were cultured with and without osteogenic induction supplemented media containing either OGL or EGL with 0.02% of IP6 for 14 days and ALP levels were estimated based on the value of absorbance measured using the microplate reader. At OGL, two-way ANOVA showed that the ‘‘treatment” factor was not significant (P = 0.13) and the ‘‘osteogenic condition” factor was significant (P < 0.001), however, the interaction between these 2 factors was significant (P = 0.003). While at EGL, the two factors and their interaction were significant (P < 0.01).

**Fig 4:**
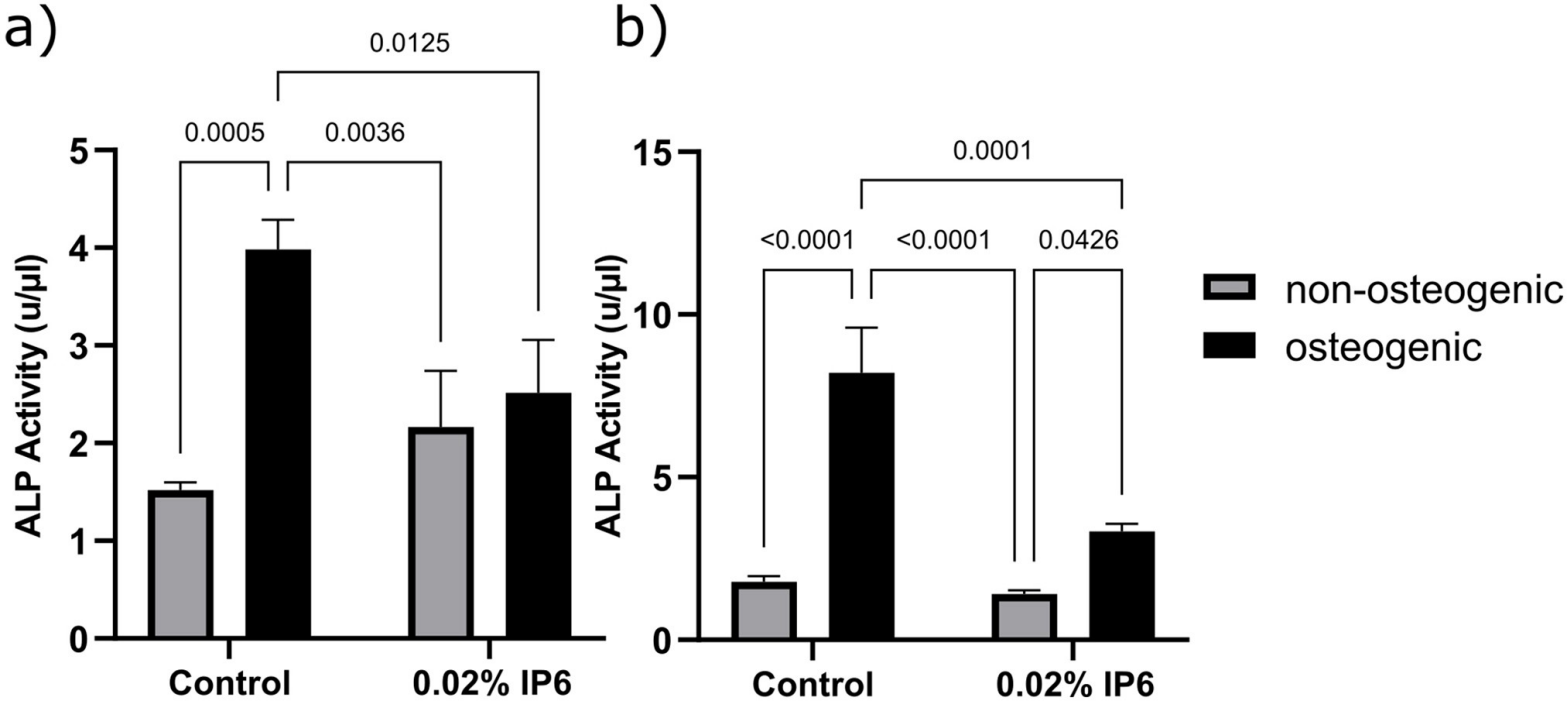
Alkaline phosphatase (ALP) activity assay using human periodontal ligament (HPDL) cells in optimal glucose level (OGL) and elevated glucose level (EGL) media under non-osteogenic and osteogenic conditions after treatment with 0.02% phytic acid (IP6) for 14 days and measured at absorbance 405 nm. **(a)** ALP activity of HPDL cells under non-osteogenic and osteogenic conditions in OGL media in control and after treatment with 0.02% IP6. **(b)** ALP activity of HPDL cells under non-osteogenic and osteogenic conditions in EGL media in control and after treatment with 0.02% IP6 for 14 days. ALP activity was quantified using a colorimetric assay and presented as mean ± standard deviation. Statistical analysis was performed using ANOVA.

The results for the ALP activity of HPDL cells in OGL and EGL media under osteogenic and non-osteogenic conditions after treatment with 0.02% IP6 are shown in Fig 4a and 4b respectively. In a culture media with OGL, control cells in an osteogenic media showed significantly higher ALP activity than control cells cultured in a non-osteogenic media (P < 0.05). In OGL and EGL media, ALP activity of the cells was not affected by the presence of IP6 in a non-osteogenic media (P > 0.05); however, in an osteogenic media IP6 lowered the ALP activity compared to the counterpart control group (P < 0.05). Cells cultured with presence of IP6 in OGL showed similar ALP activity at osteogenic and non-osteogenic media (P > 0.05); however, at EGL, IP6 within an osteogenic media resulted in higher ALP activity compared to IP6 in non-osteogenic media.

### Calcium release and alizarin red staining

HPDL cells were cultured with and without osteogenic induction supplemented media containing either OGL or EGL with 0.02% of IP6 for 21 days and the calcium release was estimated after alizarin staining. At OGL, two-way ANOVA showed that the ‘‘treatment” and ‘‘osteogenic condition” factors were significant (P<0.05), however, the interaction between these 2 factors was not significant (P = 0.65). While at EGL, the two factors and their interaction were significant (P<0.01).

**Fig 5:**
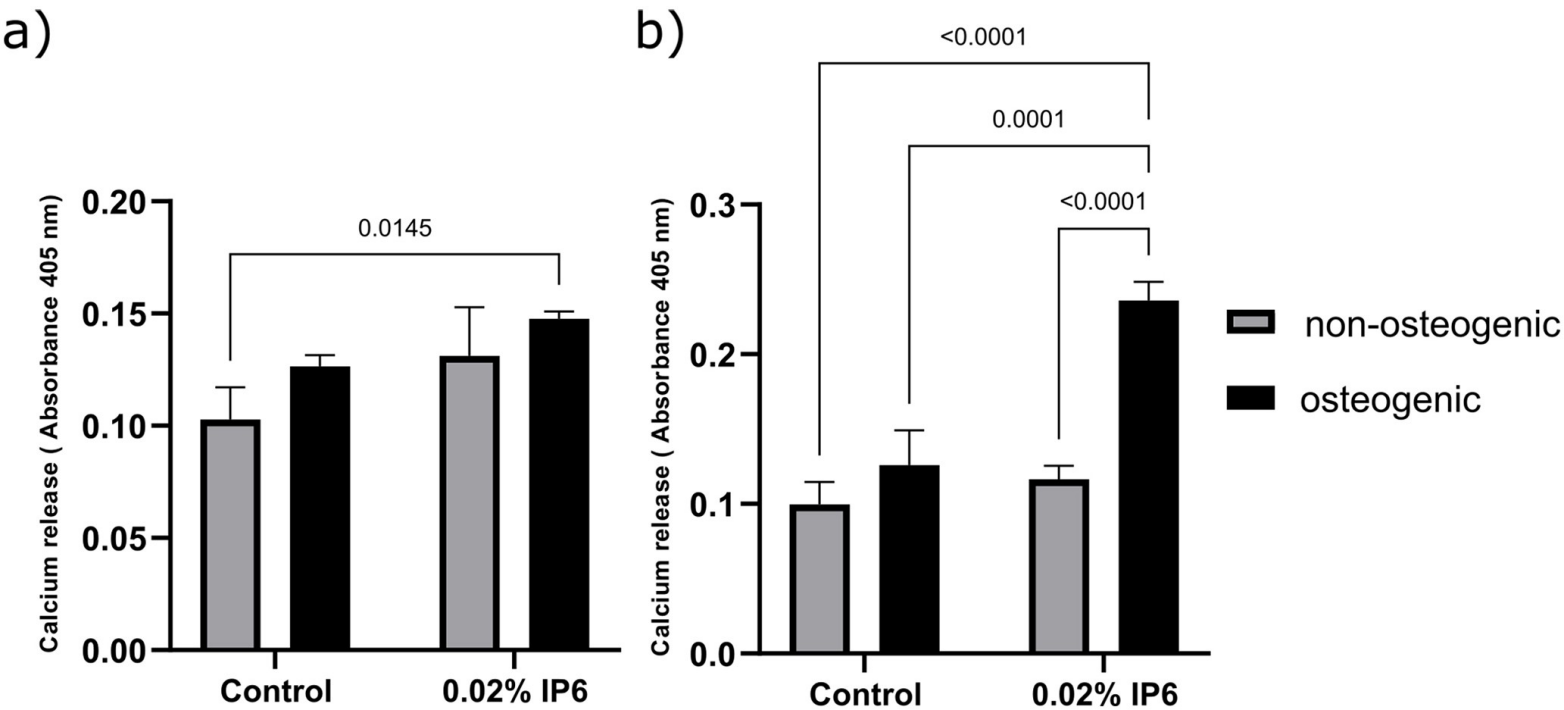
Calcium release by human periodontal ligament (HPDL) cells in optimal glucose level (OGL) and elevated glucose level (EGL) media under non-osteogenic and osteogenic conditions after treatment with 0.02% phytic acid (IP6) for 21 days and measured at absorbance 405 nm. Results are shown as mean ± standard deviation (SD). **(a)** Calcium release by HPDL cells under non-osteogenic and osteogenic conditions in OGL media in control and after treatment with 0.02% IP6. **(b)** Calcium release by HPDL cells under non-osteogenic and osteogenic conditions in EGL media in control and after treatment with 0.02% IP6. Statistical analysis was performed using ANOVA.

**Fig 6:**
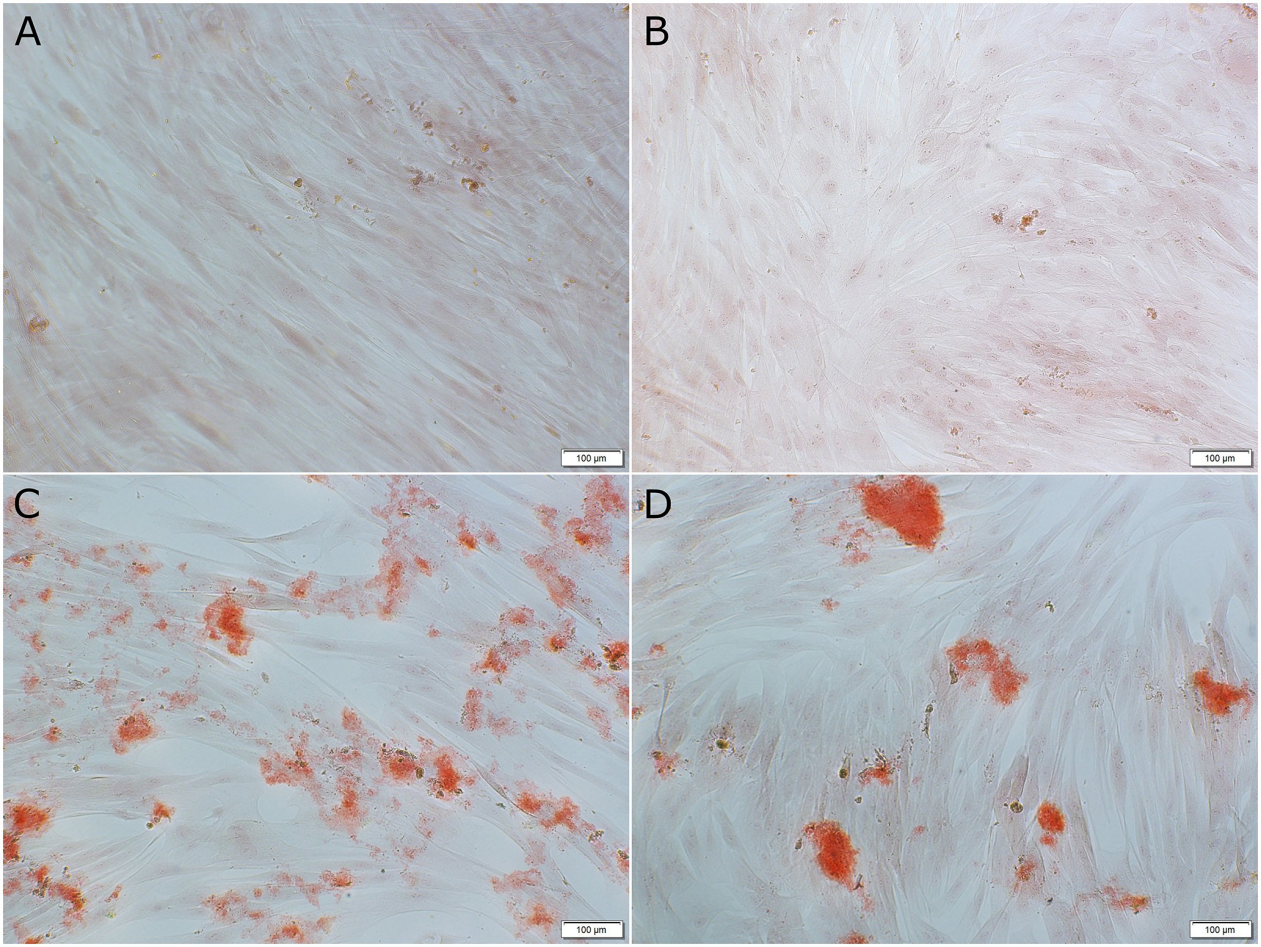
Representative images of alizarin red-stained extracellular calcium deposition in non-osteogenic and osteogenic conditions in optimal glucose level (OGL) media treated with control or 0.02% phytic acid (IP6) for 21 days. The alizarin red-stained extracellular calcium deposition is shown in red color. **(A)** control group (non-osteogenic) **(B)** control group (osteogenic) **(C)** 0.02% IP6 in (non-osteogenic) **(D)** 0.02% IP6 (osteogenic). Scale bar 100μm.

The effects of 0.02% IP6 on calcium release from HPDL cells in OGL and EGL under osteogenic and non-osteogenic conditions are shown in Fig 5a and 5b; respectively. In a media containing OGL, there was no difference between the control groups in osteogenic and non-osteogenic conditions. IP6 treated cells showed a tendency for higher calcium release in an osteogenic and non-osteogenic condition in OGL-containing media, however, this did not reach the level of significance to its counterpart control group (P > 0.05). There was also no significant difference in the results between IP6 in osteogenic condition compared to that in a non-osteogenic condition in OGL media. Representative images showing extracellular calcium deposition stained by alizarin red stain in OGL within non-osteogenic and osteogenic media for the control and 0.02% IP6 treatment are shown in Fig 6.

**Fig 7:**
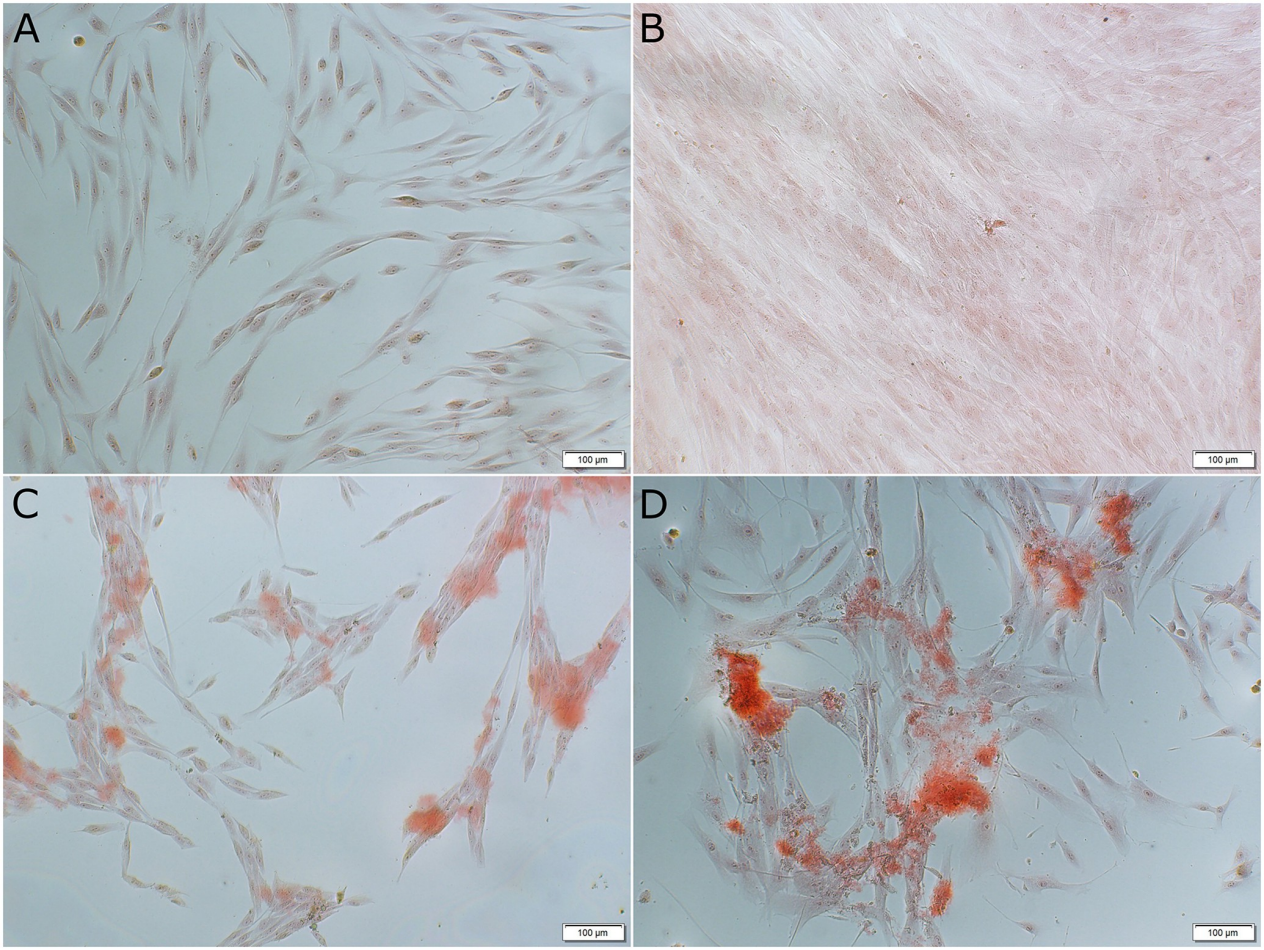
Representative images of alizarin red-stained extracellular calcium deposition in non-osteogenic and osteogenic conditions in elevated glucose level (EGL) media treated with control or 0.02% phytic acid (IP6) for 21 days. The alizarin red-stained extracellular calcium deposition is shown in red color. **(A)** control group (non-osteogenic) **(B)** control group (osteogenic) **(C)** 0.02% IP6 in (non-osteogenic) **(D)** 0.02% IP6 (osteogenic). Scale bar 100μm.

In EGL media, IP6 in an osteogenic condition resulted in a statistically higher calcium release than all other groups. In EGL media, the control group in a non-osteogenic condition showed similar calcium release to those obtained in a control group in an osteogenic media and IP6 in a non-osteogenic media (P > 0.05). Representative images showing extracellular calcium deposition stained by alizarin red stain in EGL within non-osteogenic and osteogenic media for the control and 0.02% IP6 treatment are shown in Fig 7.

## Discussions

In this study the effect of IP6 on HPDL cells was tested at two different glucose levels. The results have shown that IP6 at certain concentrations at 24 h exposure enhanced the viability of the cells at OGL while all concentrations at 48 h exposure improved the viability compared to the control. However, at EGL, all concentrations of IP6 lowered cells’ viability at both exposure times, except for 0.02% IP6 at 48 h which showed a value similar to the control. At OGL and EGL, IP6 did not affect the ALP activity of the cells in non-osteogenic media, while it decreased this activity in an osteogenic media when compared to the respective control. In OGL, calcium release was maintained with IP6 in non-osteogenic and osteogenic media. Meanwhile, IP6 at EGL increased calcium release in osteogenic media while it maintained the release in non-osteogenic media. These results require rejection of the null hypotheses.

The known antinutrient effect of IP6 by decreasing the absorption of certain minerals from food does not negate the health benefits obtained from its consumption. IP6 is considered as an antioxidant that has a role in lowering cholesterol levels and controlling blood sugar levels [22]. The direct effect of IP6 in in vitro conditions has been met with controversy. This agent was proposed to have a protective effect on normal cells through a dual mechanism; it acts as a direct source of phosphate and indirectly as an antioxidant that lowers the production of hydroxyl radicals by chelation of iron that participates in Fenton’s reaction which results in the production of free radicals [23, 24]. Certain cell culture media are known to contain elevated levels of iron [25]. We previously reported the enhanced viability of pulpal- and osteoblast-like cells in presence of IP6 [26, 27], and the present study is consistent with these findings as IP6 was found to positively impact or maintain the viability of HPDL cells in culture media with OGL at both exposure times. Moreover, at OGL, control cells showed a drop in the viability at 48 h compared to 24 h while IP6 treated cells showed a tendency of elevated proliferation. On the other hand, IP6 is also known to have anti-proliferative effect on various cancer cell lines. This inhibitory effect is the result of decreased protein synthesis and metabolic activity which reflect possible DNA-damage through a complicated interaction of IP6 with DNA. IP6 was also reported to have a proapoptotic effect on cancerous cells through increasing caspase-3 activity [28]. IP6 as any other agent has dose related multiphasic effect on cells. This hormetic effect is related to its targeted component of the cell. Pacheco et al. found that IP6 at 5 mM (0.23%) negatively affected the integrity of intestinal epithelial cell monolayers and their proliferations, while lower concentrations ameliorated cellular damage with speculated mechanisms related to the antioxidant ability of IP6 to modify the signaling pathways of the cells, detoxify reactive oxygen species or protect against lipid peroxidation [29].

Since IP6 is known for its strong antioxidant activity, and diabetes is free radicals mediated disease [17], the effect of IP6 on HPDL cells in presence of high levels of glucose was investigated. Contrary to our expectations, IP6 mostly inhibited the proliferation of HPDL cells at EGL. It is noteworthy to mention that at 48 h, there was sharp drop in the control cells viability in presence of high glucose, meanwhile IP6 maintained the viability to similar levels to those obtained at 24 h. Interestingly, at 48 h, cell viability in 0.02% IP6 treated cells was statistically similar to that of the control group. The inhibitory effect of IP6 on viability seen in the present study should not necessarily be viewed as a negative impact on the cells. The initial accelerated proliferation of cells in presence of glucose was previously reported to have deleterious effect on the long-term survival of the cells. Several molecular mechanisms are involved in cell proliferation as a response to hyperglycemia, and this include the over expression of osteopontin and the increased oxidative stress which are implicated in the pathogenesis of several systemic complication related to diabetes [17]. This might explain the finding of the present study among the control group of high glucose. The observed effect of IP6 on the cells at EGL is challenging to explain. Inositol, product of IP6 hydrolysis, and related metabolites have complex relationship with glucose at different levels. For instance, glucose and inositol share the same transporter system, and in cultured cells the latter uptake is inhibited by the presence of high levels of glucose [16]. Meanwhile, IP6 has been suggested to decrease the expression of glucose transporter GLUT-4 protein, which has a salient role in the first step of glucose utilization and uptake by the cell, thus limiting the amount of glucose that is transported into the cell resulting in cellular stress and decreased ATP levels. Moreover, increased levels of phosphorylated AMP-activated kinase was observed in presence of IP6 leading to increased AMP levels [30]. AMP levels are generally increased in hypoglycemic conditions [31]. Thus, the finding of the present study may be partly explained by IP6 ability to limit the uptake of glucose by the cells, thus preserving it to be used for longer duration of time and hence the drop in viability compared to the control especially at 24 h and the maintenance of viability at 48 h to levels compared to those obtained at 24 h.

HPDL cells are essential for the regeneration of periodontium through their contribution to the formation of bone, fibers and cementum [32]. ALP has a salient role in the formation of hard tissue, and studying its expression in presence of potential therapeutic agents provides important information on the speculated mechanisms [33]. In the present study, in an osteo control (non-osteogenic) media, IP6 showed no effect on the ALP activity of the cells compared to the control at OGL and EGL. However, in presence of osteogenic media, IP6 significantly decreased the activity and interestingly this inhibitory effect was more pronounced at EGL. It should be noted here that control cells in osteogenic media showed high levels of ALP activity especially in presence of EGL. Enhanced ALP activities are not always judged as beneficial; for instance, a positive correlation has been reported between serum glucose levels and ALP [34]. McCarthy and colleagues demonstrated that glycation end products increased ALP activity in osteoblast cells initially; however, longer duration of culturing brought the levels back to normal or decreased them [35]. Elevated levels of ALP are not always desirable; high ALP are associated with vascular calcification that has a pathogenesis closely related to serum glucose levels [36]. Thus, the observed inhibitory effect of IP6 on ALP activity in osteogenic media should be interpreted with caution as it does not necessarily eclipse the perceived benefits of this agent. The molecular basis on how IP6 affects this activity is poorly understood and various mechanisms could be ascribed for in vitro conditions. IP6 is a strong chelator of magnesium and zinc, both minerals are essential for the activity of ALP and are present in FBS to improve the culturing conditions [37, 38]. Moreover, certain components of FBS are known to contribute to mineralization and hydrolyze phosphate sources. In addition to that, FBS has an inherent ALP activity that could have influenced the data obtained in this study. Overall, the exact composition of FBS is poorly defined and not consistent and researchers are encouraged to identify the influence of certain factors within FBS on the results of their investigations. Paradoxically, the current findings showed high calcium release in presence of IP6 in osteogenic media at OGL and EGL, however, this effect was more prominent with the latter. Despite the notion that ALP is a reflection of the mineralization ability of the cells, it is not always directly proportional to the resulting mineralization [39]. Due to structural similarities between IP6 and pyrophosphate, some biological actions of the former could be better explained by findings related to the extensively studied pyrophosphate. The activities of these compounds depend on the type of tissue. For instance, in certain parts of the body, they minimize pathological calcification by chelating with calcium and preventing its precipitation [40]; while in osseous tissue, alkaline phosphatase could cleave the phosphate groups resulting in the release of phosphate and calcium which become available for the process of mineralization [41]. Indeed, IP6 has the ability to form insoluble complexes with calcium and simultaneously prevent further growth of the precipitated crystals [42]. The effect of IP6 on mineralization of cells cultures is controversial; however, Addison et al. found that despite some reports on the inhibitory effect of IP6 on certain aspects needed for mineralization, it does not impair the cells’ ability to differentiate, produce collagenous matrix, and express ALP [43]. While Arriero et al. reported conflicting findings as they used a wider range of concentrations than those with Addison et al., 0.1–100 μm versus 1–4 μm; respectively. IP6 is omnipresent in mammalian cells, with a concentration ranging from 10 to 100 μm/L [44]. Thus, concentrations used in Addison et al. and Arriero et al. studies cover mostly sub-physiological and physiological levels while those used in our study might be considered as pharmaceutical doses. The results of these two studies cannot be directly compared to the current investigation due to other reasons such as the used cell lines and origin. Moreover, the source of IP6 extraction may also affect the structure of the obtained agent and its biological activities [45]. The increased calcium deposition observed at the highest concentration of IP6 used at Arriero et al. study was assumed to be the result of IP6-calcium complexes precipitation rather than extra extracellular matrix mineralization [44]. The source of the extracellular calcified nodules seen in the present study cannot be verified.

The fundamental benefit of this study is that it was carefully planned and carried out under controlled circumstances, guaranteeing its scientific validity. Additionally, the study examined the effects of various IP6 concentrations on HPDL cells and examined those effects over a range of time periods, allowing for assessment of IP6’s dose-dependent effect.

In general, at controlled concentrations IP6 and its metabolites are considered non-toxic. Furthermore, the Food and Drug Association has granted IP6 the status “Generally Recognized As Safe”, and the European Union has approved it as a food additive [46]. Thus, we believe that IP6 warrant more attention from researchers to harness its useful properties for oral health.

## Conclusions

The limitations of this study include but are not limited to the use of in vitro cell culturing which fails to replicate the condition of the cell within its tissue of origin, thus the results of the present study do not necessarily reflect the in vivo behavior of the HPDL cells in presence of IP6. Additionally, this study only tested two different glucose levels (OGL and EGL) which might not adequately capture the wide range of glucose levels experienced in diabetic conditions. Furthermore, although the probable reasons for the observed effects are addressed, there are still questions about the precise biochemical pathways involved, which could limit the practical application of the findings. Within these limitations, IP6 enhanced or maintained the viability of the cells in presence of OGL while it mostly lowered the viability at EGL. In presence of osteogenic media, IP6 lowered the ALP activity of the cells at OGL and EGL, while it increased calcium release in osteogenic media especially with EGL. To gain a deeper insight into the therapeutic possibilities of IP6, further comprehensive investigations are essential. These encompass in vivo studies to confirm the findings and understand the practical applications of IP6. Additionally, mechanistic studies are needed to elucidate the precise effects of IP6 and its underlying mechanisms on the cells of the periodontal ligaments and the overall health of the periodontium.

## Abbreviations

ALP: Alkaline phosphatase activity
DMEM: Dulbecco’s Modified Eagle Media
EGL: Elevated glucose media
FBS: Fetal bovine serum
h: Hours
HPDL: Human periodontal ligament cells
IP6: Phytic acid
OGL: Optimal glucose media
trypsin–EDTA: 1x trypsin-ethylenediaminetetraacetic acid
